# Influence of extension stem length and diameter on clinical and radiographic outcomes of revision total knee arthroplasty

**DOI:** 10.1186/s12891-019-3030-1

**Published:** 2020-01-08

**Authors:** Sheng-Hsun Lee, Hsin-Nung Shih, Chih-Hsiang Chang, Tung-Wu Lu, Yu-Han Chang, Yu-Chih Lin

**Affiliations:** 1 Department of Orthopaedic Surgery, Chang Gung Memorial Hospital, Linkou No.5, Fuxing St., Guishan Dist., Taoyuan, 333 Taiwan (Republic of China); 2 Bone and Joint Research Center, Chang Gung Memorial Hospital, Linkou, Taiwan No.5, Fuxing St., Guishan Dist., Taoyuan, 333 Taiwan (Republic of China); 3 Department of Biomedical Engineering, National Taiwan University, Taipei, Taiwan No. 1, Sec. 4, Roosevelt Rd., Taipei, 10617 Taiwan (Republic of China)

**Keywords:** Revision total knee arthroplasty, Extension stem, Canal filling ratio, Aseptic loosening

## Abstract

**Background:**

Extension stems provide stability to revision total knee arthroplasty (RTKA). Little is known regarding the relationship between stem characteristics and RTKA stability. We aimed to identify the relationship between canal filling ratio (CFR) and aseptic loosening following RTKA.

**Methods:**

We retrospectively reviewed demographics, radiographic parameters, and outcomes associated with RTKA performed between 2008 and 2013 in a tertiary hospital. The inclusion criteria were: revision for aseptic loosening, hybrid fixation, minor bone defect, Zimmer® LCCK prosthesis, and follow-up > 24 months. Using the modified Knee Society radiographic scoring system, radiographic prosthesis loosening was defined as a radiolucent line (RLL) score ≥ 9 on the femoral side or ≥ 10 on the tibial side. We utilized receiver operating characteristic (ROC) curve analysis to evaluate the cutoff value for stem length and diameter in terms of prosthesis loosening or not. Furthermore, CFR-related parameters were analyzed with logistic regression to clarify their relationships with prosthesis loosening.

**Results:**

Prosthesis loosening was detected in 17 of 65 patients included. On logistic regression analysis, male sex and severity of the tibial bone defect were associated with loosening. On multivariate analysis, male sex and bone defect severity were associated with loosening on the femoral side, while malalignment was associated with loosening on the tibial side. Protective factors included femoral CFR > 0.85, CFR > 0.7 for > 2 cm, and CFR > 0.7 for > 4 cm, as well as tibial CFR > 0.85.

**Conclusions:**

To minimize loosening post-RTKA, femoral CFR > 0.7 for > 2 cm and tibial CFR > 0.85 are recommended. Risk factors may include male sex, bone defect severity, and malalignment.

## Background

Revision total knee arthroplasty (RTKA) is becoming increasingly common as populations age. In the United States, the annual number of RTKAs is expected to increase from 38,000 in 2005 to 268,000 by 2030 [1]. The failure mechanisms of RTKA failure are periprosthetic joint infection, aseptic loosening, instability, malalignment, and polyethylene wear [2]. One report suggested that aseptic loosening is the predominant mechanism of failure (31.2%) [3], attributed to fixation failure, insufficient reconstruction of the bone defect, or inadequate stability provided by the extension stem.

No clear principles for choosing the extension stem have been established to date. Completo et al. reported that cementless and cemented stems, respectively, share 27 and 54% of the load across the metaphyseal area [4]. Parsley et al. suggested that longer, cementless, canal-filling stems provide better tibial alignment [5], and introduced the term “canal filling ratio” (CFR) to describe the ratio between the stem diameter and medullary canal width. However, the investigation of Parsley et al. focused on RTKA alignment rather than prosthesis stability. Gililland et al. found higher failure rates post-RTKA for cementless stems engaging the diaphysis for < 4 cm than for cemented stems [6], but did not report the stem dimensions providing adequate diaphyseal engagement.

To improve our understanding of the relationship between extension stem characteristics and RTKA stability, we conducted a retrospective study based on joint registry data from a single institute. We aimed to answer the following questions: (1) What is the relationship between CFR and prosthesis stability? (2) What is the relationship between stem length and prosthesis stability? (3) What other factors contribute to prosthesis loosening?

## Methods

### Study groups

**Fig. 1:**
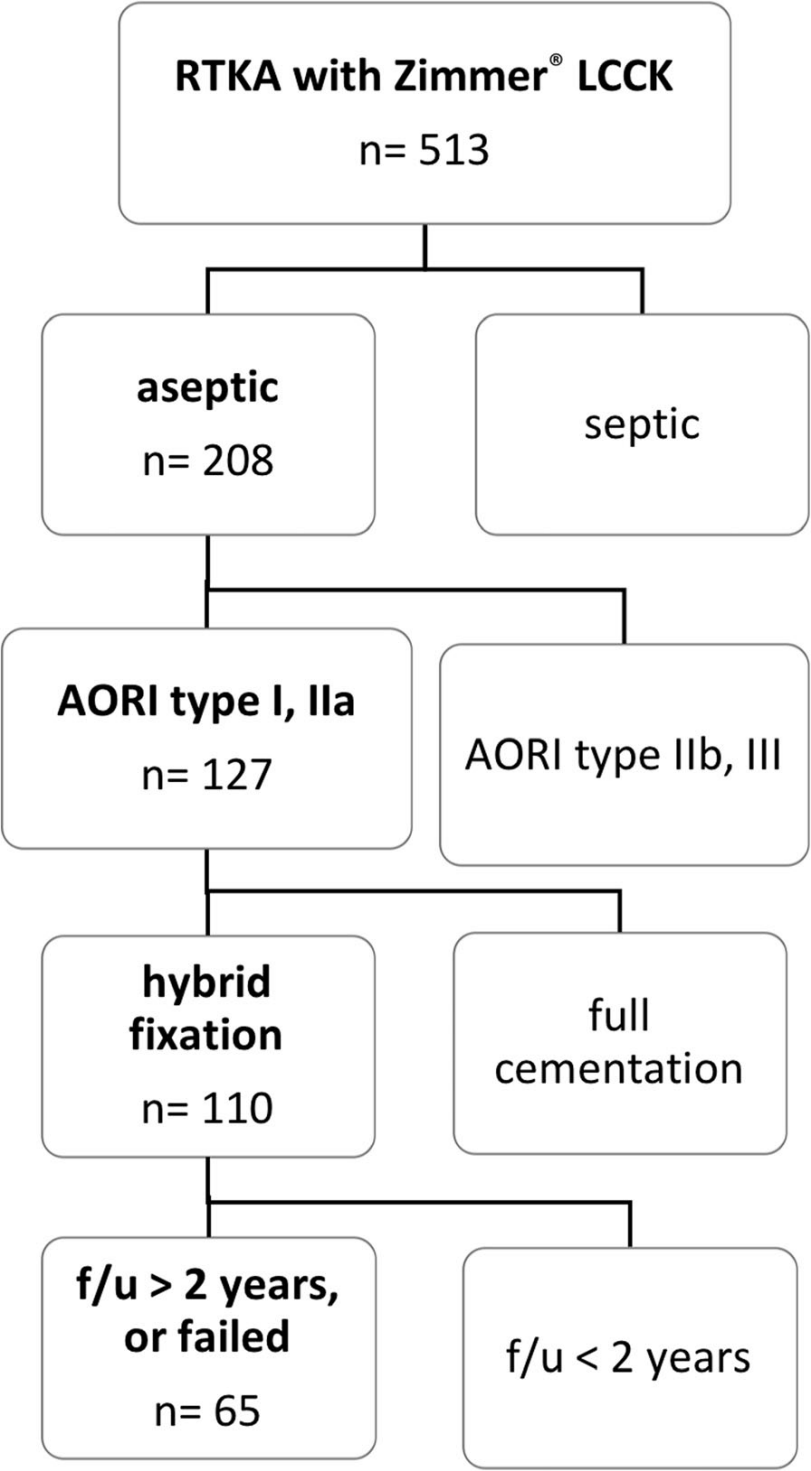
Inclusion criteria. Patients receiving RTKA with Zimmer® LCCK during 2008–2013 in our hospital were included. Revisions for periprosthetic joint infection, severe bone defects, full cementation technique, and follow-up < 2 years were excluded f/u: follow-up period. Aseptic: revision TKA due to aseptic causes.

This retrospective study used joint registry data collected and maintained by Chang Gung Memorial Hospital, Linkou Branch, which is a tertiary referral center handling > 2500 primary total joint surgeries each year. The study was conducted with approval from the hospital’s ethics review board. (IRB No.: 201801083B0) The IRB waived the requirement of informed consent. We included patients receiving RTKA with the NexGen® Legacy Constrained Condylar Knee prosthesis (LCCK; Zimmer®, Warsaw, Poland) and CCK insert between 2008 and 2013. Only patients with minor bone defects were included, i.e., defect of type I or IIa according to the Anderson Orthopaedic Research Institute (AORI) classification. Hybrid fixation was used in all patients, with cement over the epiphysis and metaphysis but not over the diaphysis. The indications for LCCK implantation were ligament insufficiency after primary total knee arthroplasty (TKA) and aseptic loosening of previous arthroplasty, including uni-compartment knee arthroplasty, TKA, and RTKA. The patient inclusion process was summarized in Fig. 1. The surgeries were performed by senior surgeons in the Joint Reconstruction Department of our hospital. We included patients with follow-up > 2 years or failed RTKA within 2 years postoperatively.

### Clinical and radiographic parameters evaluated

We collected data on demographics, bone quality, postoperative knee alignment, number and width of radiolucent lines (RLLs), CFR, prosthesis-related parameters, and clinical outcomes. Demographics included age at index surgery, sex, physical status according to the American Society of Anesthesiologists classification, body mass index (BMI), comorbidities, and Charlson comorbidity index (CCI). Radiographic evaluations were performed by two independent surgeons. Bone quality was assessed in terms of cortex thinning findings on anteroposterior and lateral radiographs, as described by Edwards et al.: good (no thinning on either radiograph), fair (thinning on one but not the other radiograph), and poor (thinning on both radiographs) [7]. Postoperative knee alignment was quantified as the femoral-tibial angle on standing scanography. Using the modified Knee Society radiographic scoring system, the radiographic stability of the knee prosthesis (stable, possibly loose requiring close follow-up, or loose, as defined by Fehring et al. [8]) was evaluated by measuring the width (in millimeters) of the RLLs in each area surrounding the prosthesis, and adding these values to obtain a score. The femoral and tibial components, respectively, are considered stable for RLL scores ≤8 and ≤ 9, possibly loose requiring close observation for scores of 9–19 and 10–22, and loose for scores ≥20 and ≥ 23. Thus, we defined loosening as an RLL score ≥ 9 for the femoral component or ≥ 10 for the tibial component.

### CFR parameters

**Fig. 2:**
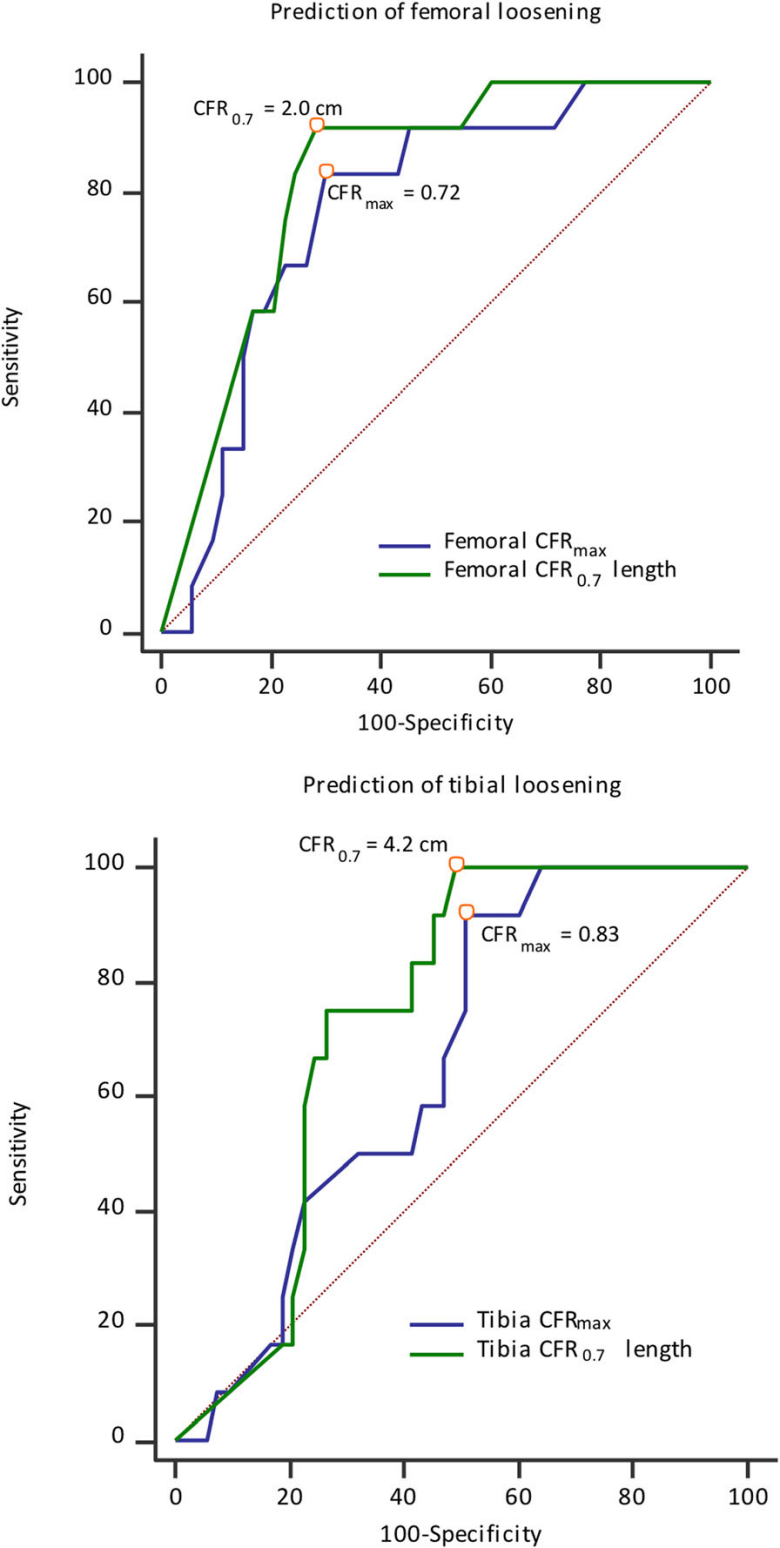
Receiver operating curve analysis for predicting prosthesis loosening. Top, femoral component. Optimal results for CFR_max_ was 0.72 (area under the curve [AUC] = 0.77), and for CFR_0.7_ length was 2 cm (AUC = 0.83). Bottom, tibial component. Optimal results for CFR_max_ was 0.83 (AUC = 0.66), and for CFR_0.7_ length was 4.2 cm (AUC = 0.74). CFR (canal filling ratio) was calculated as the ratio between the stem width and the medullary canal width. CFR_x_ length indicates the stem length for which CFR is larger than x. CFR_max_ indicates the maximum CFR over the entire length of the stem

CFR was defined as the ratio between the extension stem diameter and the medullary canal width on anteroposterior (AP) as well as lateral plain radiographs. In each knee, the CFR value we recorded was the larger one on AP or lateral radiograph. We performed receiver operating characteristic (ROC) curve analysis to determine the optimal cutoff of CFR (best sensitivity and specificity) for predicting prosthesis loosening (Fig. 2), and found maximum CFR (CFR_max_) values of 0.72 and 0.83 for the femoral and tibial side, respectively. Therefore, we used the CFR cutoffs of 0.7 and 0.85 to further evaluate the influence of stem diameter on RTKA stability. Upon combining the impact of stem diameter and length, ROC curve analysis revealed better stability for stems with CFR > 0.7 over a length of > 2 cm or > 4 cm for the femoral and tibial side, respectively.

**Fig. 3:**
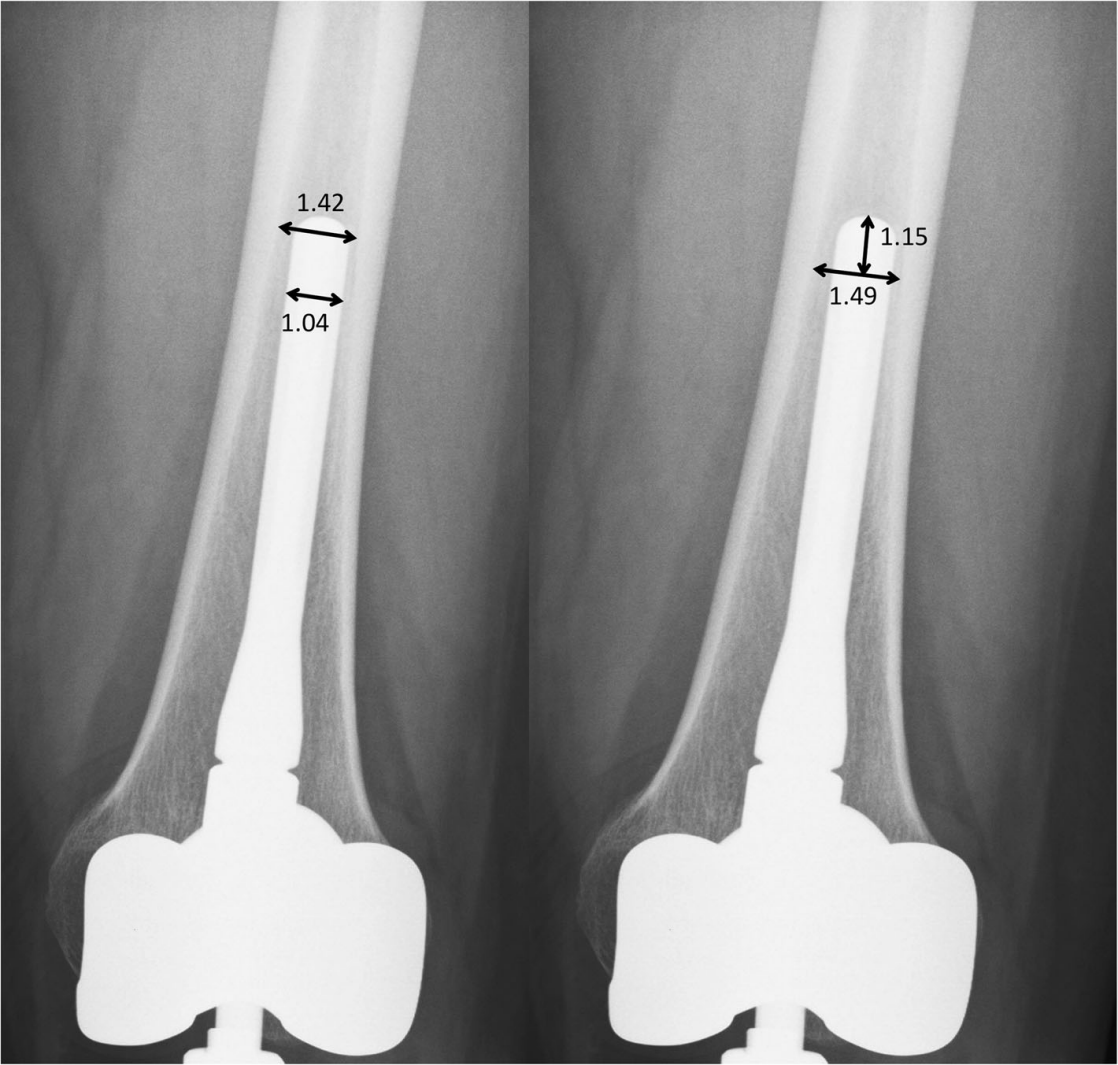
Example of CFR parameter evaluation. Left, the stem diameter is 1.04 cm, whereas the narrowest part of medullary canal which is occupied by the stem is 1.42 cm. *CFR*_*max*_ = 1.04/1.42 = 0.73. *CFR > 0.85*: negative. Right, for the calculation of *CFR*_*0.7*_ *length*, divide stem diameter (1.04 cm) by 0.7 equals to 1.49 cm. Measure the length from stem tip to the level where canal diameter is 1.49 cm. In this case, it is 1.15 cm. As a result, *CFR*_*0.7*_ *> 2 cm*: negative, *CFR*_*0.7*_ *> 4 cm*: negative*, CFR*_*0.85*_*length*: 0 cm, *CFR*_*0.7*_ *length*: 1.49 cm

We then analyzed the following CFR-related parameters with univariate analysis to evaluate the risk factors of prosthesis loosening (Fig. 3):

1. Factors of stem diameter: any part of the stem which has CFR > 0.85 (CFR_0.85_), maximal CFR (CFR_max_);
2. Factors of stem diameter and length: CFR > 0.7 over a certain length of the stem (CFR_0.7_ > 2 cm and CFR_0.7_ > 4 cm), stem length for which CFR > 0.85 (CFR_0.85_ length), and stem length for which CFR > 0.7 (CFR_0.7_ length).

We did not evaluate CFR_0.7_ (any part of the stem which has CFR > 0.7) because it was a rather loose criterion which we thought had little discriminating power. Instead, we combined the effect of length and diameter to see whether the length of particular part the stem where CFR > 0.7 was longer than 2 cm (CFR_0.7_ > 2 cm) or 4 cm (CFR_0.7_ > 4 cm). Similarly, the particular part of stem length where CFR > 0.7 (CFR_0.7_ length) or 0.85 (CFR_0.85_ length) was evaluated. Significant risk factors were then proceeded to multivariate analysis.

Patients were followed-up per our routine protocol for arthroplasties, at 6 weeks, 6 months, and one year postoperatively, and bi-annually thereafter. Clinical outcome was quantified as the Knee Society Score (KSS), which was evaluated by residents or nurse practitioners before the index surgery and either at final follow-up or before re-RTKA.

### Statistical analyses

Standard descriptive statistics included mean, range, and frequency. Normality was checked by Kolmogorov-Smirnov test. Comparison of baseline characteristics was carried out by chi-square and independent *t*-test for nominal and continuous variables, respectively.

We used ROC curve analysis with DeLong’s method to determine the CFR cutoff. Logistic regression was used to evaluate potential risk factors for loosening in univariate analysis. The independent *t*-test was used to compare KSS data. Multivariate analysis with adjusted odds ratios was performed using the stepwise method. We used Kaplan-Meier survival analysis with the endpoint of radiographic loosening to depict the prosthesis survivorship when individual CFR parameter was met or not.

MedCalc Statistical Software version 17.9 (MedCalc Software bvba, Ostend, Belgium) was used for statistical analyses. A *p*-value < 0.05 was considered to indicate statistical significance.

## Results

Of the 65 patients (65 knees) included in the study, 17 had loosening, while 48 did not (Table 1). Four patients with loosening required re-RTKA. Male sex was more common among patients with loosening than among those without (52.9% vs. 18.8%, *p* = 0.007). Mean age, BMI, physical status, Charlson index, and bone quality did not differ significantly between the two groups. More severe bone defects on the tibial side (AORI type IIa) were more common in patients with loosening than among those without (*p* = 0.010), while no such difference was observed for the femoral side. Regarding the type of prosthesis before the index surgery, patients with previous RTKA tended to have a higher incidence of loosening, but this tendency did not reach statistical significance. No intra-operative fractures occurred during prosthesis implantation.

**Table 1:** Baseline demographic and clinical characteristics

Characteristic	Loosening (*n* = 17)	No loosening (*n* = 48)	*p*
Age, years	69.22 (52–79)	71.94 (56–86)	0.553
Female sex	47.06 (8)	81.25 (39)	0.007^*^
BMI	27.99 (21.77–41.09)	28.35 (21.35–37.76)	0.748
ASA score ≥ 3	52.94 (9)	50.00 (24)	0.828
CCI			
1–2	29.41 (5)	29.17 (14)	0.413
3–4	70.59 (12)	56.25 (27)	
≥ 5	0	14.58 (7)	
Bone quality			
Femur (loosening, *n* = 12)			
good	0 (0)	5.66 (3)	0.498
fair	66.67 (8)	73.58 (39)	
poor	33.33 (4)	20.75 (11)	
Tibia (loosening, n = 12)			
good	8.33 (1)	1.89 (1)	0.437
fair	50.00 (6)	62.26 (33)	
poor	41.67 (5)	35.85 (19)	
Bone defect severity			
Femur (loosening, *n* = 12)			
I	25.00 (3)	52.83 (28)	0.113
IIa	75.00 (9)	47.17 (25)	
Tibia (loosening, *n* = 12)			
I	8.33 (1)	49.06 (26)	0.010^*^
IIa	91.67 (11)	50.94 (27)	
Prosthesis before revision			
Primary TKA	82.35 (14)	89.58 (43)	0.092
UKA	0 (0)	6.25 (3)	
Revision TKA	17.65 (3)	2.08 (1)	
None	0 (0)	2.08 (1)	
Follow-up, months	80.11 (33.15–137.53)	67.73 (24.02–131.58)	0.091

Data are shown as frequency (number) or mean (range) unless otherwise specified. Bone defect severity was assessed using the Anderson Orthopaedic Research Institute classification. *ASA* American Society of Anesthesiologists; *BMI* body mass index; *CCI* Charlson comorbidity index; *TKA* total knee arthroplasty; *UKA* uni-compartment knee arthroplasty; *: *p* < 0.05

ROC curve analysis revealed an optimal CFR_max_ of 0.72 for the femoral side (sensitivity, 83.3%; specificity, 69.8%; area under the curve [AUC], 0.77) and 0.83 for the tibial side (sensitivity, 91.7%; specificity, 49.1%; AUC, 0.66) (Fig. 2). The optimal stem length with CFR > 0.7 was 2.0 cm for the femoral side (sensitivity, 91.7%; specificity, 71.7%; AUC, 0.83) and 4.2 cm for the tibial side (sensitivity, 100%; specificity, 50.9%; AUC, 0.74). Therefore, we further evaluated the frequency of CFR_0.85_, CFR_0.7_ > 2 cm, and CFR_0.7_ > 4 cm as factors potentially associated with prosthesis stability.

Loosening was noted for 12 femoral components and 12 tibial components, including 7 patients with loosening of both components (Table 2). On univariate analysis, CFR_0.85_, CFR_0.7_ > 2 cm, CFR_0.7_ > 4 cm, CFR_max_, CFR_0.85_ length, and CFR_0.7_ length were all associated with femoral component stability, whereas all parameters except for CFR_max_ and CFR_0.85_ length were significantly associated with tibial component stability. Male sex and bone defect of AORI type IIa were significantly associated with prosthesis loosening. Regarding radiographic findings, knee alignment differed significantly between the two groups, with a median femoral-tibial angle of 5.0° (interquartile range [IQR], 2.1°–6.2°) in patients with loosening and 5.8° (IQR, 5.2°–7.4°) in those without (*p* = 0.006).

**Table 2:** Univariate analysis of risk factors for aseptic loosening after revision total knee arthroplasty

Potential risk factor	Loosening (*n* = 17)	No loosening (*n* = 48)	OR	95% CI	*p*
Age, years	69.22	71.94	0.95	0.87–1.02	0.188
Female sex	8	39	4.88	1.47–16.13	0.009^*^
Bone defect severity					
Femur (loosening, *n* = 12)					
I	3	28	0.29	0.07–1.22	0.075
IIa	9	25			
Tibia (loosening, *n* = 12)					
I	1	26	0.09	0.01–0.78	0.005^*^
IIa	11	27			
Prosthesis before revision					
Primary TKA	14	43	2.28	0.86–6.01	0.094
UKA	0	3			
Revision TKA	3	1			
None	0	1			
Stem length, mm					
Femur	100 (75–200)	100 (25–200)	0.990	0.96–1.01	0.990
Tibia	75 (25–150)	100 (25–200)	0.98	0.96–1.01	0.157
Radiographic finding					
Alignment, °	5.0 (2.1–6.2)	5.8 (5.2–7.4)	0.62	0.43–0.87	0.006^*^
Femur (loosening, *n* = 12)					
CFR_0.85_	8.33 (1/12)	52.83 (28/53)	0.08	0.01–0.67	0.020^*^
CFR_0.7_ > 2 cm	16.67 (2/12)	75.47 (40/53)	0.07	0.01–0.33	0.001^*^
CFR_0.7_ > 4 cm	8.33 (1/12)	45.28 (24/53)	0.11	0.01–0.91	0.041^*^
CFR_max_	0.69 (0.54–1.00)	0.82 (0.47–1.00)	0.94	0.89–0.98	0.010^*^
CFR_0.85_ length	0.0 (0.0–4.7)	1.2 (0.0–7.8)	0.37	0.13–0.97	0.045^*^
CFR_0.7_ length	0.0 (0.0–7.5)	3.5 (0.0–10.3)	0.50	0.31–0.79	0.003^*^
Tibia (loosening, *n* = 12)					
CFR_0.85_	8.33 (1/12)	47.17 (25/53)	0.10	0.01–0.84	0.034^*^
CFR_0.7_ > 2 cm	41.67 (5/12)	77.36 (41/53)	0.21	0.05–0.77	0.020^*^
CFR_0.7_ > 4 cm	16.67 (2/12)	58.49 (31/53)	0.14	0.02–0.71	0.018^*^
CFR_max_	0.77 (0.60–0.88)	0.82 (0.53–1.00)	0.96	0.91–1.01	0.159^*^
CFR_0.85_ length	0.0 (0.0–1.7)	0.0 (0.0–12.8)	0.40	0.15–1.07	0.069^*^
CFR_0.7_ length	1.6 (0.0–4.3)	4.5 (0.0–4.7)	0.70	0.52–0.94	0.019^*^

Data are shown as frequency (number/total) or median (interquartile range) unless otherwise specified. Bone defect severity was assessed using the Anderson Orthopaedic Research Institute classification. Alignment indicates the femoral-tibial angle. CFR was calculated as the ratio between the stem width and the medullary canal width. CFR_x_ stands for CFR > x. CFR_x_ > y indicates the stem length > y for CFR larger than x. CFR_x_ length indicates the stem length for which CFR > x. CFR_max_ indicates the maximum CFR over the entire length of the stem. CI, confidence interval; CFR, canal filling ratio; OR, odds ratio; TKA, total knee arthroplasty; UKA, uni-compartment knee arthroplasty; *: *p* < 0.05

**Fig. 4:**
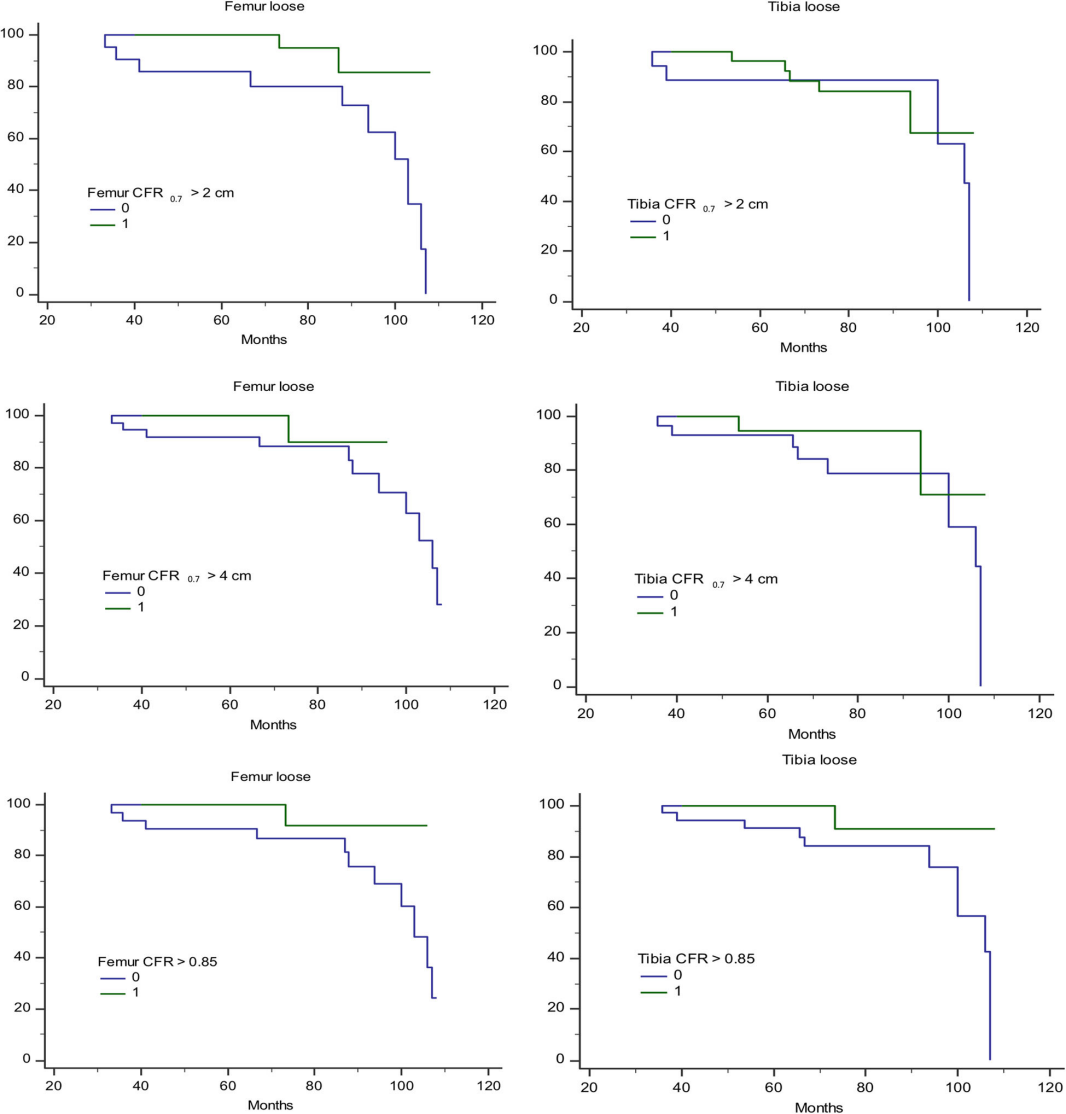
Kaplan-Meier survival curves describing survival free from prosthesis loosening. Left, femoral component. CFR_0.7_ > 2 cm, CFR_0.7_ > 4 cm, and CFR_0.85_ were all associated with less loosening. Right, tibial component. Only CFR_0.85_ was associated with less loosening CFR (canal filling ratio) was calculated as the ratio between the stem width and the medullary canal width. CFR_x_ > y indicates stem length > y for which CFR is larger than x.

On multivariate analysis, male sex and bone defect severity were associated with femoral but not tibial component loosening (Table 3). Femoral component stability remained associated with CFR_0.85_, CFR_0.7_ > 2 cm, CFR_0.7_ > 4 cm, CFR_0.7_ length, and CFR_0.85_ length, whereas tibial component stability was only associated with knee alignment and CFR_0.85_. Kaplan-Meier survival analyses for specific CFR parameters were summarized in Fig. 4.

**Table 3:** Multivariate analysis of risk factors for aseptic loosening after revision total knee arthroplasty

Femur						Tibia				
Potential risk factor	Crude OR	95% CI	Adjusted OR	95% CI	*p*	Crude OR	95% CI	Adjusted OR	95% CI	*p*
Age, years	0.96	0.87–1.04	0.95	0.82–1.10	0.513	0.94	0.86–1.02	0.94	0.81–1.08	0.421
Female sex	5.35	1.41–20.12	6.75	1.04–43.45	0.045^*^	3.42	0.92–12.55	2.67	0.38–18.80	0.323
Bone defect severity	0.30	0.07–1.22	0.10	0.01–0.82	0.032^*^	0.09	0.01–0.78	0.01	0.00–2.85	0.103
Prosthesis before revision	3.11	1.13–8.53	1.21	0.34–4.29	0.766	3.11	1.13–8.53	2.29	0.53–9.74	0.262
Radiographic finding										
Alignment	0.73	0.55–0.97	0.99	0.65–1.48	0.958	0.57	0.39–0.83	0.49	0.27–0.88	0.017^*^
CFR_0.85_	0.08	0.01–0.67	0.06	0.01–0.68	0.024^*^	0.10	0.01–0.84	0.07	0.01–0.80	0.032^*^
CFR_0.7_ > 2 cm	0.07	0.01–0.33	0.02	0.01–0.33	0.006^*^	0.21	0.05–0.77	0.33	0.06–1.72	0.188
CFR_0.7_ > 4 cm	0.11	0.01–0.91	0.04	0.01–0.66	0.025^*^	0.14	0.02–0.71	0.23	0.03–1.48	0.123
CFR_max_	0.94	0.89–0.98	0.93	0.87–1.00	0.051	0.96	0.91–1.01	0.55	0.32–0.90	0.407
CFR_0.85_ length	0.37	0.13–0.97	0.36	0.12–1.00	0.050^*^	0.40	0.15–1.07	0.38	0.13–1.09	0.073
CFR_0.7_ length	0.50	0.31–0.79	0.43	0.23–0.79	0.007	0.70	0.52–0.94	0.76	0.55–1.03	0.081

Bone defect severity was assessed using the Anderson Orthopaedic Research Institute classification. Alignment indicates the femoral-tibial angle. CFR was calculated as the ratio between the stem width and the medullary canal width. CFR_x_ stands for CFR > x. CFR_x_ > y indicates stem length > y for CFR larger than x. CFR_x_ length indicates the stem length for which CFR > x. CFR_max_ indicates the maximum CFR over the entire length of the stem. CI, confidence interval; CFR, canal filling ratio; OR, odds ratio; *: *p* < 0.05

The two groups did not differ significantly regarding clinical, functional, or total scores preoperatively (Table 4). However, patients with loosening had lower functional and total scores postoperatively (*p* = 0.021 and *p* = 0.043, respectively). Two patients without loosening reported pain in the tibial midshaft (pain scores, 3 and 4 on the numerical rating scale), and both had tibial stem CFR > 0.85.

**Table 4:** Knee Society Score evaluating the outcomes of revision total knee arthroplasty

Score	Loosening	No loosening	*p*
Pre-OP			
Clinical	40 (25–53)	47 (28–56)	0.323
Functional	54 (22–63)	51 (19–59)	0.558
Total	98 (51–116)	102 (49–112)	0.337
Post-OP			
Clinical	75 (67–83)	81 (73–90)	0.089
Functional	62 (53–72)	83 (70–91)	0.021^*^
Total	135 (122–153)	166 (149–178)	0.043^*^

Data are shown as mean (range). Pre-OP, before revision total knee arthroplasty; Post-OP: before re-revision surgery in patients with prosthesis loosening, or at final follow-up in those without loosening. *: *p* < 0.05

## Discussion

In this retrospective study, we evaluated the correlation between extension stem characteristics and prosthesis stability following RTKA with the same type of prosthesis (Zimmer® LCCK) and fixation technique (hybrid fixation) in patients with minor bone defect (AORI type I/IIa). We tried to set up a principle of stem choice, by which surgeons can estimate the optimal CFR and stem length in preoperative templating or intraoperative trial. We found that the optimal choice of extension stem was CFR_0.85_ > 0 cm or CFR_0.7_ > 2 cm for the femoral component, and CFR_0.85_ > 0 cm for the tibial component. Male sex and bone defect of AORI type IIa were associated with femoral component loosening, while varus alignment was associated with tibial component loosening.

To date, there are no clear guidelines regarding the choice of extension stem in RTKA. Parsley et al. reported better alignment for CFR > 0.85 [5], while Gililland et al. reported good stability for diaphyseal engagement > 4 cm without defining diaphyseal engagement or specifying the reason behind choosing this cutoff [6]. To our knowledge, the present study is the first to evaluate the potential association of RTKA stability with extension stem diameter and length. The various CFR-based criteria evaluated here were identified based on ROC curve analysis, which indicated CFR > 0.72 and CFR > 0.85 as predictors of prosthesis loosening on the femoral and tibial side, respectively. We found that not only the diameter of the stem, but also its length can be important for prosthesis stability. Indeed, CFR_0.7_ > 2 cm and CFR_0.7_ > 4.3 cm were also predictors of femoral and tibial component stability, respectively.

Our results suggest that CFR_0.7_ > 2 cm is sufficient to ensure femoral component stability, while tibial component stability requires CFR > 0.85, which might imply that the tibial component is more prone to loosening. Previous observations in this respect are highly discrepant. Leta et al. reported that loosening was almost twice more likely to occur at the tibial than at the femoral side [2]. Fehring et al. reported higher loosening rate at the femoral side, while others found similar loosening rates for the femoral and tibial components [6, 8, 9]. The discrepancy in these findings might be related to bone defect severity. Specifically, we only included knees with minor defects (AORI type I/IIa), which likely provided relatively good bone-prosthesis contact in the femoral box area, resulting in improved stability. In addition, the femoral component is intrinsically more stable than the tibial component because of the “pinching” effect provided by the anterior flange and posterior condyle of the femoral prosthesis, which is known to affect the stability of cementless TKA [10, 11]. Previous studies on primary TKA also reported more aseptic loosening at the tibial than at the femoral side [12, 13]. Thus, the femoral stem may not need a very high CFR to achieve good stability.

Regarding stem design, we believe that a porous (rather than polished) stem will provide better initial stability and promotes bone incorporation later on, ensuring long-term durability. However, inserting or extracting porous stems is more technically demanding. To exclude the effect of stem design when evaluating the impact of stem diameter and length, we only included patients with the same prosthesis type and stem design.

Regarding RTKR prosthesis fixation, the hybrid approach typically involves using a longer diaphyseal-engaging stem with cement fixation over the epiphyseal and metaphyseal areas, whereas fully cemented fixation often involves using a shorter stem with cementation of the entire prosthesis-bone interface. Fehring et al. reviewed 475 RTKAs and reported more RLLs for hybrid than for fully cemented fixation [8], while Greene et al. reported no aseptic loosening on midterm follow-up of 119 RTKAs with hybrid fixation [14]. Similarly, Edwards et al. reported less radiographic loosening for hybrid-fixation RTKA involving a two-stage exchange arthroplasty protocol for periprosthetic joint infection [7]. Edwards et al. also found that hybrid fixation was not associated with increased infection rate despite using less antibiotic-loaded cement. In their radiostereometric analysis, Heesterbeek et al. found no fixation-related difference in prosthesis micromotion following RTKA [15]. Because there is ongoing controversy regarding which type of fixation provides better stability, we only included RTKA with hybrid fixation, to exclude the effect of such confounding factors when assessing the influence of stem characteristics on stability.

Adequate reconstruction of the bone defect is key for long-term stability of the knee prosthesis. Various methods for reconstruction of more severe bone defects have been reported (e.g., allograft, cone, sleeve), with good clinical outcomes [16–18]. A stable initial environment for incorporating the bone graft can be achieved using the extension stem, which can provide prosthesis stability, ensure correct alignment, and spread the loading of the prosthesis-bone junction, thus protecting the bone graft from early failure [19]. Completo et al. confirmed that the stem can spread the loading across the bone graft-cement interface, on both the femoral and tibial side [4, 20]. In the current study, multivariate analysis revealed bone defect severity (AORI type IIa) as a risk factor for loosening at the femoral but not the tibial side. While increased defect severity understandably compromises prosthesis stability, the AORI classification is somewhat arbitrary, and the distinction between type I and type IIa may be subjective (high inter-observer disagreement). Therefore, the effect of bone defect severity on prosthesis stability may not be as substantial in patients with mild defects, which could also explain why we found no correlation between tibial bone defect and tibial loosening. No other reports have indicated that AORI type I and IIa defects would have a different effect on implant stability. A previous study on RTKA treated AORI type I and IIa defects as a single category [6]. To exclude the effect of very high defect severity, we included only patients with type I/IIa defects.

We found that male sex may be a risk factor for loosening of the femoral component after RTKA, which is similar to previous observations in a Norwegian joint registry [2]. It was hypothesized that higher BMI, more intense use of the prosthetic joint, and malalignment may contribute to early loosening. To the best of our knowledge, no study has evaluated the relationship between knee alignment and RTKA failure, although many have assessed the relationship between alignment and primary TKA loosening. Ritter et al. found that, following primary TKA, the risk of failure is lowest (0.6%) for an overall alignment of 3°–7° valgus and highest for more varus or valgus alignment (1.5 and 1.4%, respectively) [21]. Fang et al. concluded that, in primary TKA, the varus knee tends to fail due to medial tibial collapse, while the valgus knee tends to fail due to ligament instability [22]. In our series, the median overall alignment was 5.0° (IQR, 2.1°–6.2°) and 5.8° (IQR 5.2°–7.4°) among patients with and without loosening, respectively. Most patients with loosening had varus malalignment.

Fracture around the stem can occur when using a stem with loo large a diameter. Cipriano et al. found an incidence of 1 and 4.9% for femur and tibia periprosthetic fracture, respectively, among 634 press-fit stems [23]. All but one such fractures were treated conservatively (one received a cable wire), and all healed uneventfully, without implant loosening. Fortunately, there were no fractures in our series, possibly due to the relatively small sample size. We assessed outcomes using Knee Society scores. Patients with loosening had significantly worse functional scores and total scores, while the clinical scores were only slightly worse. Hardeman et al. also reported slightly worse clinical scores and functional scores in patients with RLL score ≥ 4 versus < 4 [24]. However, an RLL score ≥ 4 might not necessarily indicate prosthesis loosening, since inter- and intra-observer variability may be substantial for such a small cutoff. Therefore, in consideration of the modified Knee Society radiographic scoring system, we defined loosening as an RLL score ≥ 9 for the femur and ≥ 10 for the tibia component [8].

Pain around the stem tip is often noted for stemmed prostheses used in total hip arthroplasty or revision knee arthroplasty. Two of our patients reported shin pain around the tip of the tibial stem, and both had CFR > 0.85. The symptoms were mild and resolved with oral analgesics. Stem tip pain might reflect a mismatch of elastic modulus between the stem and cortical bone [25]. In total hip arthroplasty, such pain could be resolved by plating around the stem tip, a technique later adopted in RTKA [25, 26]. The stem material may also play an important role in stem tip pain. Peters et al. reported an incidence of only 2% for stem tip pain after RTKA with fluted titanium stems [27], while Barrack et al. reported an incidence of 18.8% for solid fluted CoCr stems and 8.1% for slotted titanium stems [28]. In the present series, we used the Zimmer LCCK prosthesis with a titanium stem, which might explain the low incidence of stem tip pain. However, when planning to use stems with larger CFR, the possibility of stem tip pain is non-negligible and should be explained to the patient preoperatively.

We further discuss study limitations other than the retrospective design. First, the retrospective study design make it difficult to set up a definitive guideline for stem choice. Rather, we attempted to understand the importance of CFR by these clinical data. We will try to further prove the results through finite element and biomechanical study in the future. Second, as in other studies [6–8], we evaluated prosthesis stability on plain X-ray and used the Knee Society radiographic scoring system to avoid detection of non-significant micromotion. However, this approach might underestimate micromotion. Third, evaluation of bone quality (good, fair, or poor) was based on an arbitrary method [7]. Future studies should apply a more objective approach for assessing bone quality. Fourth, we did not assess collateral ligament status and thus could not exclude the effect of collateral ligament competence on stress loading at the prosthesis-bone junction. Nevertheless, it has been demonstrated that collateral ligaments contribute little to the varus-valgus stability of knee joints with LCCK prostheses, as this design limits ligament elongation [29].

## Conclusions

Protective factors for prosthesis loosening include CFR > 0.85 or CFR > 0.7 for > 2 cm for the femoral component, and CFR > 0.85 for the tibial component. Male sex and bone defect severity (AORI type IIa) were associated with femoral loosening, while more varus alignment was associated with tibial loosening. In RTKA, proper choice of extension stem diameter and length minimizes radiographic loosening.

## Supplementary information

**Additional file 1:.** raw data. The excel file incorporates the raw data of the patients included in the present study.

## Data Availability

The datasets used and analyzed during the current study are accessible in Additional file 1.

## Abbreviations

AORI: Anderson Orthopaedic Research Institute
ASA: American Society of Anesthesiologists
AUC: Area under the curve
BMI: Body mass index
CCI: Charlson comorbidity index
CFR: Canal filling ratio
KSS: Knee Society Score
RLL: Radiolucent line
ROC: Receiver operating characteristic
RTKA: Revision total knee arthroplasty
TKA: Total knee arthroplasty
UKA: Uni-compartment knee arthroplasty
